# Relationships between Serum Levels of Atazanavir and Renal Toxicity or Lithiasis

**DOI:** 10.1155/2015/106954

**Published:** 2015-05-07

**Authors:** C. I. Marinescu, M. Leyes, M. A. Ribas, M. Peñaranda, J. Murillas, A. A. Campins, L. Martin-Pena, B. Barcelo, C. Barceló-Campomar, F. Grases, G. Frontera, Melchor Riera Jaume

**Affiliations:** ^1^Infectious Disease Service, Son Espases Hospital, Carretera de Valldemossa 79, Palma de Mallorca, 07010 Illes Balears, Spain; ^2^Multidisciplinary Group for Infectious Disease Service, Institute of Health Sciences Research, IdISPa, Health Research Foundation Ramón Llull (FISIB), Son Espases Hospital, Carretera de Valldemossa 79, Building “S”, 1st Floor, Palma de Mallorca, 07010 Illes Balears, Spain; ^3^Clinical Analysis Service, Son Espases Hospital, Carretera de Valldemossa 79, Palma de Mallorca, 07010 Illes Balears, Spain; ^4^Pharmacy Service, Son Espases Hospital, Carretera de Valldemossa 79, Palma de Mallorca, 07010 Illes Balears, Spain; ^5^Laboratory of Renal Lithiasis Research (IUNICS-IDISPa), Universitat de les Illes Balears, Carretera de Valldemossa, km 7.5, Palma de Mallorca, 07010 Illes Balears, Spain; ^6^Clinical Trials Unit, Son Espases Hospital, Carretera Valldemossa 79, Palma de Mallorca, 07010 Illes Balears, Spain

## Abstract

The main aim of this study is to describe the relationship between serum levels of atazanavir, renal toxicity, and lithiasis. This is a prospective observational study of patients being treated with atazanavir (ATV) at Son Espases Teaching Hospital, Palma de Mallorca, between 2011 and 2013. The study includes 98 patients. Sixteen were found to have a history of urolithiasis. During a median monitoring period of 23 months, nine patients suffered renal colic, in three of whom ATV crystals were evidenced in urine. Cumulative incidence of renal colic was 9.2 per 100 patients. The variables related to having renal colic were the presence of alkaline urine pH and lower basal creatinine clearance. The mean serum level of ATV was slightly higher in patients with renal colic—1,303 *μ*g/L versus 1,161 *μ*g/L—but did not reach statistical significance. Neither were any significant differences detected by analysing the levels according to the timetable for ATV dosage. Cumulative incidence of renal colic was high in patients being treated with ATV, in 33% of whom the presence of ATV crystals was evidenced in urine. We were unable to demonstrate a relationship between ATV serum levels and renal colic or progression towards renal failure.

## 1. Introduction

Highly Active Antiretroviral Treatments (HAART) are evolving very quickly; currently, there are 24 antiretroviral drugs. Belonging to six families, they offer us the possibility of being able to establish multiple personalised therapies for each patient. The toxicity of antiretroviral drugs in the medium and long term is a limiting factor which makes it necessary to modify the antiretroviral regimen in 30–45% of patients [1].

Atazanavir (ATV), one of the antiretroviral drugs approved by the FDA in June 2003, is an azapeptide that inhibits the action of HIV protease (PI). Its pharmacokinetic profile allows administration once a day with or without boosting with ritonavir, although the most widespread use in Europe is boosted with ritonavir ATV/r. Even though ATV evidenced an excellent safety profile in pivotal studies, with good gastrointestinal tolerance and few adverse effects, it produces a 4% increase in patients' bilirubin, and alterations in cardiac conduction, exacerbation of diabetes mellitus, and nephrolithiasis have been described [2, 3]. Although ATV is primarily metabolised by the liver, approximately 7% of ATV/r is excreted in urine unmetabolised, and its solubility decreases when urine alkalinity rises [4]. Observational studies reveal an increased incidence of lithiasis in patients treated with ATV compared to other antiretroviral regimens [5, 6].

Observational studies and long-term monitoring of some clinical trials also appear to show that cumulative exposures to tenofovir (TDF) and PIs such as ATV and indinavir (IDV) increase the incidence of Chronic Kidney Disease (CKD), especially in patients with other comorbidities such as hypertension (HT) and diabetes mellitus [7–9]. Many of the proposed mechanisms of nephrotoxicity due to antiretroviral drugs are related to proximal tubular damage due to mitochondrial toxicity, or to tubulointerstitial damage due to the formation of crystals. However, the pathogenic mechanism whereby the joint administration of ATV and TDF increases the incidence of CKD is not well established.

The main aim of the present study was to determine, on a prospective basis, the incidence of renal lithiasis and CKD in patients being treated with atazanavir and establish the relationship between ATV levels and the development of renal lithiasis.

## 2. Material and Methods

This is a prospective observational study in which all HIV-infected patients who attended as outpatients in the Infectious Diseases Service of Son Espases Hospital, on treatment with ATV during the last 6 months, were invited to participate.

Patients were included consecutively from February 2011 to February 2012 and were monitored until June 2013.

All the patients participating in the study signed the informed consent form, and the protocol was passed by the Balearic Islands Research Ethics Committee (CEI).

The following clinical-epidemiological variables were extracted from a specific database (eVIHa): age, gender, anthropometric data (weight, height, and body mass index (BMI)), risk group (HIV infection risk factors, date of HIV diagnosis, basal HIV stage according to the classification of the Center for Disease Control (CDC), and the start date for ATV therapy), laboratory determinations (markers of progression of HIV infection, creatinine, creatinine clearance, and urine sediment), months on ART, previous and current ARTs, comorbidities, clinical evolution and changes in ART during the monitoring period, and reason for changing the regimen.

A questionnaire was performed at the beginning of the study concerning the timetable for taking ATV, history of lithiasis, urological treatments for lithiasis, consumption of fizzy drinks, citrus fruits, vegetarian diets, and concomitant treatments that could alkalinize urine.

During the monitoring period of the study, the following were assessed on each visit: episodes of renal colic since starting ATV therapy (diagnosed in a medical service or with the presence of irradiated lower back pain, dysuria, and/or vegetative symptoms accompanied by haematuria and/or radiographic abnormalities), episodes of hepatitis, and changes in concomitant treatments.

Analytical data were collected at the beginning of atazanavir therapy, basal (date consent was signed), and prospectively every 6 months until completion of the two-year monitoring period of the study.

Biomarkers of HIV-1 activity were analysed: viral load (VL), log VL, and CD_4_ lymphocytes; blood biochemistry values: plasma creatinine, clearance of creatinine with the formulas of Cockcroft-Gault and MDRD (Modification of Diet in Renal Disease), total bilirubin, alanine aminotransferase (ALT), and aspartate aminotransferase (AST), phosphorous, and uric acid; and urine sediment: proteinuria, glycosuria, and leukocyturia. Urine pH and crystalluria were also determined in fresh morning urine.

ATV serum levels were determined using High-Performance Liquid Chromatography (HPLC) in the routine extractions for analysis.

Renal failure was considered creatinine clearance (ClCr) <60 mL or a reduction in creatinine clearance >20 mL.

Statistical analyses were carried out with the IBM SPSS Statistics 20.0 programme. Continuous variables with normal distribution are described using the mean and standard deviation, while nonnormally distributed variables are expressed as median and quartiles. Qualitative variables are described using frequency and percentage. Comparison of proportions between qualitative variables was performed using the Chi-squared statistical measure. For the comparison between quantitative variables, Student's *t*-test was used; for nonnormally distributed quantitative variables, Mann-Whitney's *U* test was used. A bivariate analysis of the variables related to renal function deterioration was performed. In all the statistical analyses, an error probability of *α* < 0.05 was considered significant.

## 3. Results

A total of 98 patients diagnosed with HIV infection being treated with atazanavir accepted to be included, out of the 248 receiving this treatment at our hospital. The median monitoring period was 23 months, between February 2011 and June 2013. The characteristics of the patients included are shown in Table 1. Seventy-two were male (73.5%) and 26 female (26.5%); the mean age was 46.1 years, ranging between 26 and 76 years. The mean weight was 68.2 kg (SD = 15.6) with a BMI of 24.2 (SD = 4.4).

Sixteen patients had a history of urolithiasis at the beginning of the study. No differences were observed in history of renal lithiasis according to gender (*p* = 0.88), age, or BMI (*p* = 0.196).

**Figure 1:**
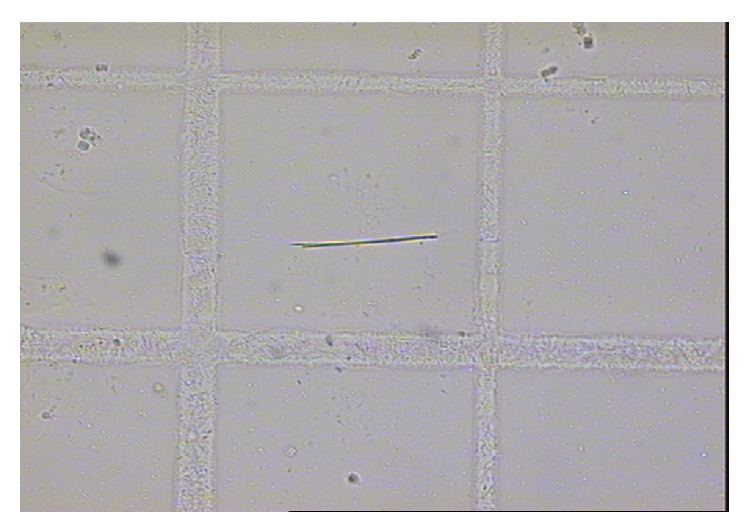
Atazanavir crystal in urine (optical microscopy) (1 × 200 *μ*m).

**Figure 2:**
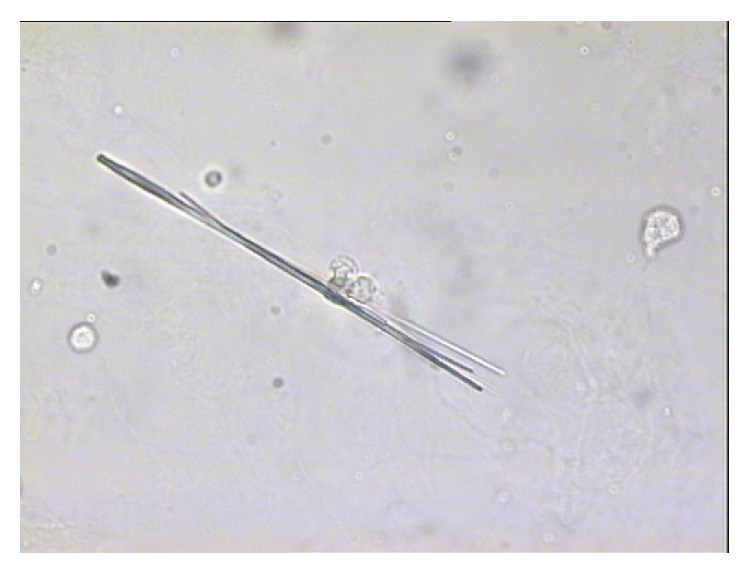
Atazanavir crystal in urine (optical microscopy) (1 × 500 *μ*m).

**Figure 3:**
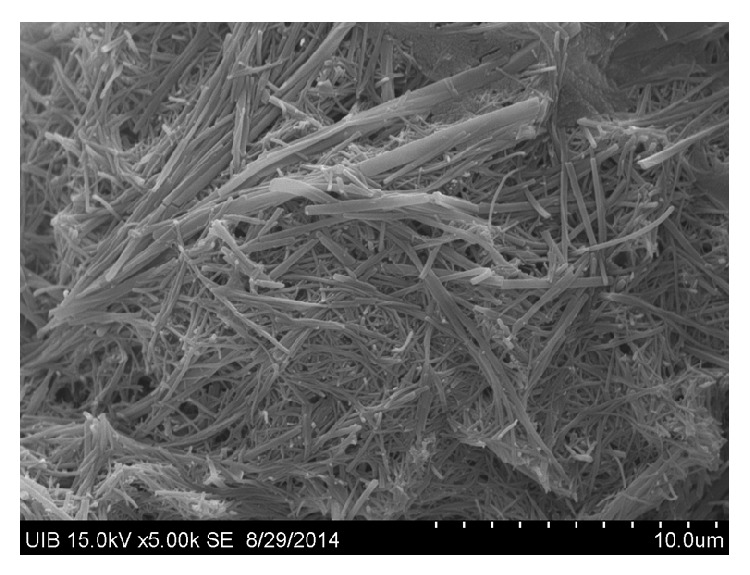
Atazanavir crystal in urine (electron microscopy) (1 × 20 *μ*m). 60 mg/L of atazanavir of urine was crystallized in 12-well plates at 37°C. It is a sample of the precipitate that forms on the bottom, where the filamentary structure is seen.

**Figure 4:**
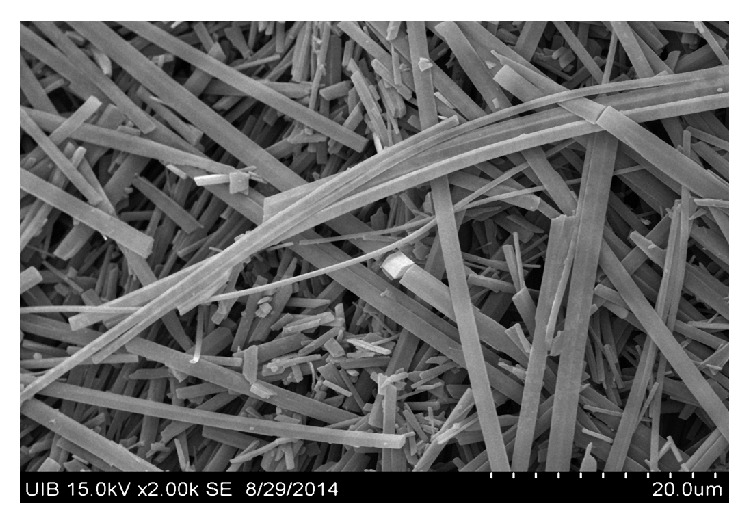
Atazanavir crystal in urine (electron microscopy) (10 *μ*m). 60 mg/L of atazanavir in urine in glass was crystallizing precipitator (turbidimeter) at 37°C. At the end, it was capped with parafilm and stored for 3 weeks.

Of the patients included in the study, nine (9.18%) had renal colic during the monitoring period, with a cumulative incidence of 9.2 per 100 patients in a median of 23.3 months monitoring period and an incidence rate of 0.053 years^−1^. Two patients had three episodes of renal colic and one patient had two. Eight of the nine patients with colic required emergency hospital care. Four patients complained of urinary urgency with dysuria and polaquiuria, seven had lower back pain, and five had nausea and vomiting. The characteristics of patients who had renal colic are compared with those who did not in Table 2. Of the 9 patients with renal colic, the presence of crystals in urine was found in six (66.6%); this turned out to be ATV in three patients and amorphous urate in the other three (see Figures 1, 2, 3, and 4). In patients without renal colic, crystals were observed in 15 (17%); these were calcium oxalate in six and urate in nine. No relationship was observed between suffering an episode of renal colic and a previous history of lithiasis. Urine pH of patients with renal colic was significantly more alkaline: 6.08 versus 5.58 (*p* = 0.048). The mean serum level of ATV was slightly higher in patients with renal colic, 1,303 *μ*g/L versus 1,161 *μ*g/L, but did not reach statistical significance. Neither were any significant differences detected by analysing the levels according to the timetable for ATV dosage; see Table 2. We also did not observe differences in the levels of atazanavir according combos used.

Criteria of chronic renal failure were found in 12 patients (16.2%) at the end of the study: creatinine clearance (ClCr) <60 mL (5 patients) or a reduction in creatinine clearance >20 mL (7 patients). A bivariate analysis of the variables related to renal function deterioration is shown in Table 3. Patients who developed renal failure were older (*p* < 0.034) and had a history of diabetes mellitus *p* < 0.0001 (OR 15, CI 95% 2.35–95.4) and hypertension *p* = 0.002 (OR 8.14, CI 95% 1.8–35.3), a tendency to present more advanced stages of the disease, stage C (CDC) *p* = 0.086 (OR 2.9, CI 95% 0.83–10.4), poorer virological control with high log VL levels (*p* < 0.001), and lower CD4 lymphocytes (*p* = 0.076). Renal failure was not related to basal renal function before starting atazanavir, a previous history of lithiasis, or the presence of renal colic during the monitoring period. No relationship was observed between the drop in ClCr and ATV levels.

During the study, two patients being treated with atazanavir who suffered toxic hepatitis were identified. ATV was discontinued in another eight patients due to renal toxicity, in six due to lithiasis, in one because of kidney failure, and in another because of virological failure and renal deterioration.

## 4. Discussion

The present study shows, in a prospectively followed cohort of patients, that the incidence of renal colic in patients being treated with atazanavir is high: 9.2 per 100 patients, and atazanavir crystals are evidenced in a third of the symptomatic patients. Renal lithiasis and the resulting renal colic are the main cause of change from ATV to other antiretroviral therapies.

The incidence of renal colic due to ATV has been studied previously in retrospective cohorts in two centres, revealing significant differences of 23 per 1000 person/year found by Hamada in Japan as opposed to the 7.3 (CI 95% 4.7–10.8) described by Rockwood in Great Britain [5, 6]. The definition of renal colic was quite different in both articles: in Rockwood only patients with radiographic criteria of renal lithiasis or obstructive uropathy were included. The incidence of 9.2 per 100 patients found in our prospective study is much higher than previous studies, using clinical-radiographic criteria similar to the ones used by Hamada, perhaps as a result of its prospective nature or due to other environmental differences. The atazanavir and lamivudine simplification study, ATLAS, conducted in Italy evidenced the presence of lithiasis in 10% of patients included, relating this with the greater levels of ATV reached after withdrawal of tenofovir (TNV) from the ART [10, 11]. The articles by Rockwood and Hamada compared the incidence of renal colic in patients who had received ATV as opposed to other regimens. In Rockwood's, the adjusted incidence rate was 5.67 (CI 95% = 3.60–9.36) per 1000 patients/year with ATV as opposed to 1.51 (CI 95% = 0.85–2.40) per 1000 patients/year among those treated with efavirenz (EFV) or other PIs [6]. In Hamada's study the incidence was ten times higher in the group treated with ATV compared to the group treated with other IPs, HR 10.44; CI 95% = 3.685–29.6 [5]. A subsequent study by Nishijima et al. observed that the incidence of nephrolithiasis in the group treated with ATV/r was 20.2 times higher than in patients treated with darunavir (DRV) [12].

Over 80% of kidney stones are produced by calcium oxalate or calcium phosphate, whereas the ones produced by uric acid, cysteine, or struvite (ammonium-magnesium phosphate) are less frequent [13–15]. Among our patients, crystalluria was mainly due to amorphous urates and calcium oxalate, although three of the nine patients (33%) with symptomatic renal colic had atazanavir crystals.

The pathogenic mechanism to explain the greater incidence of renal colic in patients with ATV is not clearly established [11]. It has been postulated that as what happens in indinavir (IDV) calculi, precipitation of ATV in the renal tubules may be the main cause, and this may be the consequence of the fact that 7% of ATV and 20% of IDV are excreted unmetabolised in urine, unlike other PIs. A recent study by de Lastours et al. observed a greater concentration of ATV and DRV in urine than in plasma, whereas lopinavir (LPV) urine levels were comparable [16]. Atazanavir solubility in urine and the time to crystallization decrease, as urine is alkalized at pH > 6, and the ATV urine concentration surpasses 30 mg/L, as was observed in our center. One of the significant variables related to the presence of renal colic in our study was to have alkaline urine with a pH > 6. The described cases of intratubular ATV precipitation confirmed by renal biopsies [17] and the presence of ATV crystals in urine in three patients who had renal colic would advocate this. With indinavir, our group showed that it is possible to reduce the crystallization of the drug in urine with the use of aescin, a compound which delays crystallization [18].

In our study we were not able to demonstrate that renal lithiasis is related to significantly higher mean levels of atazanavir, although the levels were slightly higher in patients who had recently taken it (drug dosages at lunch and dinner time). Neither were we able to demonstrate a greater increase of bilirubin in patients with renal calculi. The study by Hamada et al. revealed significant differences in bilirubin levels in patients when they had renal colic, suggesting that individuals with a slow metabolism of ATV/r, and therefore with higher levels of the drug, would have a greater incidence of lithiasis [5].

Recently the Nishijima group showed in a multivariate analysis a significant association between ATV induced nephrolithiasis and changes in the single-nucleotide polymorphisms at positions c211, 339, and 440 in the UDP-glucuronosyltransferase 1A-3′ region [19].

Discontinuance of ATV therapy in 24 patients, in 10 of them due to toxicity, was somewhat higher than expected, as ATV in previous cohort studies was shown to be one of the better tolerated drugs over a long term [20].

Out of 98 patients, at the end of the study, 12 met criteria of renal failure, but no relationship was found with ATV levels or with the presence of renal colic during the monitoring period. Variables related to renal function deterioration older age, presence of diabetes, HTA, bad control of the viral infection, and low CD4 lymphocytes were similar to those described in our eVIHa all cohort [21] and previously in studies on European cohorts [9, 22].

*Strengths and Limitations of the Present Study.* The main strength of the present study is its prospective character, with sample collection, and its design to identify all the episodes of renal colic within a fairly homogeneous cohort of patients.

The main limitation is the number of patients included and a higher-than-expected discontinuance of ATV therapy (due to simplification and toxicity), which reduced the expected observation period. ATV dosage at different times could also mean a limitation when aiming to compare levels of the drug; however, the data are shown according to the time of dosage.

## 5. Conclusions

The incidence of renal colic in patients being treated with atazanavir is high, greater than in previous observational studies; we found evidence of atazanavir crystals in a third of symptomatic patients, and renal lithiasis was the main cause of change from ATV to other antiretroviral drugs.

It is possible to adopt measures that will decrease the incidence of renal lithiasis due to ATV: by increasing liquid intake, decreasing urine alkalinisation (by avoiding fizzy drinks, severe vegetarian diet, citrate containing foods, and antacids), or acidifying the urine (by cranberry intake). Possibly avoiding supratherapeutic levels of ATV in patients with pharmacogenetic predisposition or finding substances that will inhibit its crystallization in urine could help in the future.

## Figures and Tables

**Table 1:** Characteristics of the patients in the study.

Patient variables included	*n* = 98
Gender (M/F) *n* (%)	72 (73.5%)/26 (26.5%)
Age (years) mean (SD)	46.1 (8.4)
Patient-year at risk	168.4
HIV risk group (*n* (%))	
UDVP	30 (30.6)
Homosexual	27 (27.6)
Heterosexual	32 (32.6)
Unknown	9 (9.2)
HIV stage (*n*)	
Stage A (A1/A2/A3)	2/23/13
Stage B (B1/B2/B3)	4/8/14
Stage C (C1/C2/C3)	1/6/27
Grouped BMI *n* (%)	
<20	18 (18.4)
20–30	70 (71.4)
>30	9 (9.2)
Unknown	1 (1.0)
HIV infection duration, years (mean) (SD)	16.3 (6.5)
Basal CD4, cells/*µ*L (median) (1Q–3Q)	573 (350–795)
Basal HIV RNA (log_10_ cop/mL) mean (SD)	2.5 (1.1)
Basal HIV RNA <50 copies/mL (%)	90.0%
Cumulative ATV exposure, months (median) (1Q–3Q)	19.2 (9.5–37.5)
Time of ATV dosage (B/L/D) (%)	30.1/20.4/49.5
Administration of TDF in ART (%)	72.0%
Bilirubin at onset of ART/basal (mg/dL)	0.7/1.7
ClCr by MDRD at onset of ART/basal (SD)	98.0 (25.0)/94.7 (22.4)
History of lithiasis *n* (%)	
Yes (mean)	16 (16.3)
No (mean)	74 (75.5)
Unknown (mean)	8 (8.2)
Atazanavir combo (%)	
TDF/FTC (tenofovir/emtricitabine)	64.0
LMV/ABV (lamivudine/abacavir)	15.4
Others	20.6

*n*: number; M/F: male/female; SD: standard deviation; UDVP: intravenous drug use; BMI: body mass index; B/L/D: breakfast, lunch, and dinner; ClCr: creatinine clearance; MDRD: Modification of Diet in Renal Disease.

**Table 2:** Characteristics of patients who had renal colic are compared to those of who did not have renal colic.

	Renal colic	Without renal colic	*p* value
	(*n* = 9)	(*n* = 89)	
Gender (M/F) *n* (%)	7/2 (77.8/22.2)	65/24 (73.03/26.97)	0.81
Mean age (years)	46.2	45.2	0.74
Grouped BMI (<20/20–30/>30) *n* (%)	4/4/1 (44.4/44.4/11.2)	14/66/9 (15.73/74.16/10.11)	0.66
Basal CD4 lymphocytes, median (cells/*μ*L)	706	547	0.27
Basal VL (log_10_cop/mL) (mean)	1.7	2.5	0.07
Cumulative ATV exposures (months)	25.5	25.9	0.95
Lithiasis before start of study (*n*) (%)	2 (22.22)	14 (15.73)	0.74
Basal renal function by MDRD (mL/min/1.73 m^2^)	82.2	99.9	0.03
Basal uric acid (mg/dL)	2.9	0.4	0.22
Increase in bilirubin (mg/dL) (mean)	0.42	0.70	0.74
Type of crystalluria (total) (%)	66.6	16.8	<0.001
Calcium oxalate *n* (%)	0 (0)	6 (6.9)	
Amorphous urate *n* (%)	3 (27.3)	9 (10.3)	
ATV *n* (%)	3 (27.3)	0 (0)	
Urine pH	6.1	5.6	0.048
Time of ATV dosage (B/L/D)	3/3/3	25/16/42	0.77
Mean levels of ATV (overall) (*µ*g/L)	1303	1161	0.67
B dosage patient levels	368	594	0.55
L dosage patient levels	1312	834	0.12
D dosage patient levels	1921	1578	0.52

*n*: number; M/F: male/female; BMI: body mass index; MDRD: Modification of Diet in Renal Disease; B/L/D: breakfast, lunch, and dinner.

**Table 3:** Characteristics of patients who had renal failure at the end of the study compared to those who did not have renal failure.

	Renal failure			
	Yes (*n* = 12)	No (*n* = 62)	*p* value	OR (confidence interval) for renal failure/CI (at 95% level)
Gender (M/F) (*n* = 74) (%)	9/3 (75.0/25.0)	44/18 (70.97/29.03)	0.930	a
Age (years)	51.17	45.3	0.034	1.074 (1.002–1.150)
Grouped BMI (*n* = 74)	24.8	24.3	0.73	a
HIV risk group (*n* = 74) (%)				
HTSX	7 (58.33)	18 (12.90)	0.049	3.42 (0.96–12.2)
Non-HTSX	5 (41.67)	44 (87.10)		
Lithiasis before start/after end of study (*n* = 67) (%)	3/7 (30/70)	10/47 (17.54/85.46)	0.358	a
Renal colic before start/after end of study (*n* = 72) (%)	1/10 (9.09/90.91)	4/57 (6.56/93.44)	0.76	a
Crystalluria after onset of ATV (*n* = 74) (%)	3/9 (25.0/75.0)	10/52 (16.13/83.87)	0.103	a
Stage C HIV (yes/no) (*n* = 74) (%)	5/7 (41.67/58.33)	20/42	0.086	2.99 (0.8–10.9)
Diabetes mellitus (yes/no) (*n* = 74) (%)	4/8 (33.33/66.67)	2/60 (3.23/96.77)	0.0001	15 (2.4–95.4)
HTA (yes/no) (*n* = 74) (%)	5/7 (41.67/58.33)	5/57 (8.06/91.94)	0.002	8.14 (1.8–35.3)
Overall levels of ATV (*µ*g/L)	1074	1237	0.55	a
Basal CD4 (cells/*µ*L)	345	574.4	0.072	0.998 (0.995–1.000)
Basal viral load (Log_10_ copies/mL)	3.96	2.42	0.002	2.46 (1.37–4.40)
Basal Cl creatinine MDRD (mL/min/1.73 m^2^)	94.5	98.7	0.56	a

*n*: number; CI: confidence interval; M/F: male/female; BMI: body mass index; HTSX: heterosexual; HTA: high blood pressure; MDRD: Modification of Diet in Renal Disease; a: odds ratio (OR) cannot be calculated for risk groups.
